# nRCFV: a new, dataset-size-independent metric to quantify compositional heterogeneity in nucleotide and amino acid datasets

**DOI:** 10.1186/s12859-023-05270-8

**Published:** 2023-04-12

**Authors:** James F. Fleming, Torsten H. Struck

**Affiliations:** University of Oslo Natural History Museum, Sars’ Gata 1, Oslo, Norway

**Keywords:** Phylogenetics, Compositional heterogeneity, Bioinformatics software

## Abstract

**Motivation:**

Compositional heterogeneity—when the proportions of nucleotides and amino acids are not broadly similar across the dataset—is a cause of a great number of phylogenetic artefacts. Whilst a variety of methods can identify it post-hoc, few metrics exist to quantify compositional heterogeneity prior to the computationally intensive task of phylogenetic tree reconstruction. Here we assess the efficacy of one such existing, widely used, metric: Relative Composition Frequency Variability (RCFV), using both real and simulated data.

**Results:**

Our results show that RCFV can be biased by sequence length, the number of taxa, and the number of possible character states within the dataset. However, we also find that missing data does not appear to have an appreciable effect on RCFV. We discuss the theory behind this, the consequences of this for the future of the usage of the RCFV value and propose a new metric, nRCFV, which accounts for these biases. Alongside this, we present a new software that calculates both RCFV and nRCFV, called nRCFV_Reader.

**Availability and implementation:**

nRCFV has been implemented in RCFV_Reader, available at: https://github.com/JFFleming/RCFV_Reader. Both our simulation and real data are available at Datadryad: https://doi.org/10.5061/dryad.wpzgmsbpn.

## Introduction

Addressing many evolutionary questions requires knowledge of how the investigated taxa are related to one another. As such, phylogenies must be reconstructed [1, 2]. These reconstructions should be as accurate as possible despite model inadequacies and data paucity. Modern phylogenetic reconstruction depends on the application of explicit substitution models with specific assumptions about the underlying molecular evolutionary processes and their statistical properties [3, 4]. For example, most of these models, including those in common use, assume that the evolutionary process is Markovian and stationary, implying that the sequences are likely to be compositionally homogeneous at any point in time [5–8]. Sometimes, this assumption is violated by large amounts of change occurring along one or several edges in a tree, or by directionally selective amino acid changes that alter the tertiary structure of the protein. These violations may manifest themselves differently in phylogenetic analyses, ranging from erroneously estimated edge lengths or poorly supported bipartitions, to topological changes grouping taxa together solely based on shared composition. These effects have been widely reported and studied for many years [5–7, 9–17]. In some cases, but not all, these studies showed that these violations are also linked to long branch attraction artefacts [10]. Hence, compositional heterogeneity is a widespread and challenging problem within phylogenetic analyses and should be accommodated [6, 10, 17–19]

One common approach to address the problem of compositional heterogeneity is to identify which genes, partitions and taxa do not fulfil the assumptions of compositional homogeneity or stationarity and then exclude these from phylogenetic reconstructions to investigate what effect this exclusion has on the phylogenetic reconstruction [19]. In this approach, a statistical comparative measurement of compositional heterogeneity is applied across taxa and genes or partitions. The Relative Composition Variation (RCV) was one of the first of these measurements [20], though the RxC Chi-square test [21] was already available to compare between taxa. However, while the RCV metric is normalised over the number of taxa, the value is based on the number of actual occurrences of states and hence is influenced by changes in the length of the sequences in the dataset. As such, a comparison across both taxa and partitions is challenging, because RCV is not independent of the number of positions and so does not exclusively quantify variation in composition. To overcome this problem, a metric called Relative Compositional Frequency Variation (RCFV) was proposed and implemented in BaCoCa [22, 23]. Instead of number of occurrences, it uses the relative frequencies of the characters in the data. Therefore, it should not be susceptible to changes in sequence length and number of positions. This makes it a powerful tool when applied to large or otherwise computationally demanding phylogenetic datasets, and it can by itself act as an early-warning sign for compositional heterogeneity. Accordingly, it has been applied in many studies, including large-scale phylogenomic analyses [12, 24–29]. The data matrices generated from these measurements can then be further explored using different statistical approaches and significance tests based on the preferences of the user [23].

RCFV is a relatively simple calculation that compares the relative frequency of a given nucleotide or amino acid for a given taxon versus the mean relative frequency of the same nucleotide or amino acid over the entire dataset, expressed as follows:

1 $$RCFV=\sum_{i=1}^{n}\sum_{j=1}^{j=m}\frac{\left|{\mu }_{ij}-\overline{{\mu }_{j}}\right|}{n}$$

with *n* being the number of taxa and *j* = *1* to *j* = *m* being the possible character states—for example: 4 for nucleotide data and 20 for amino acid data. μ_ij_ represents the relative frequency of character *j* in sequence *i*, and $\overline{{\mu }_{j}}$ is the average relative frequency of character *j* across the entire dataset. This means that a higher RCFV is more indicative of compositional heterogeneity within the dataset than a small RCFV [22]. These values can then be calculated for character-specific (csRCFV, Eq. 2) and taxon-specific (tsRCFV, Eq. 3) variations to better assess the variability of these factors within a dataset for the purposes of excluding potentially heterogeneous taxa or characters [23].

2 $$csRCFV=\sum_{i=1}^{n}\frac{\left|{\mu }_{ij}-\overline{{\mu }_{j}}\right|}{n}$$

3 $$tsRCFV=\sum_{j=1}^{j=m}\frac{\left|{\mu }_{ij}-\overline{{\mu }_{j}}\right|}{n}$$

These values, thereby, do not only comprise individual taxa and character states, but also monophyletic groups of taxa or character states (e.g., purines vs pyrimidines). Within the RCFV framework, csRCFV and tsRCFV can be assessed as divisions of the total RCFV. This means that through a simple division, the user can assess the percentage RCFV that a single taxa or character contributes to the total dataset. For example, in a given nucleotide dataset, if RCFV is 0.1, and csRCFV(A) is 0.025, then A is contributing 25% of the total RCFV score. This ought to be expected in a dataset where compositional heterogeneity is not dependent on over or under-representation of a single character.

Phylogenetic datasets grow more and more in both length and depth in response to both broader and deeper sampling and increasing computational power. More data calls for a more rigorous selection of the data that is included in actual analyses, through excluding data strongly violating the assumptions of the applied models of reconstructions [30]. However, this requires that the selection criteria assess what they are supposed to assess and that they are not influenced by other aspects of the dataset. As mentioned above, the normalization over the number of taxa and the usage of relative frequencies should theoretically render RCFV values independent of the number of taxa and positions. However, this theoretical assumption has never been tested, especially over the large datasets that are now commonplace in phylogenetics in the 2020s. Only if this assumption is correct can RCFV guarantee that taxa or partitions are excluded based on variation in composition, and not in the number of taxa and/or positions in the alignment.

RCFV-type metrics have an important place in understanding compositional heterogeneity, that makes them unique, and complementary, to other approaches, such as matched-pair tests [17]. When compared to alternative tests, RCFV allows researchers to explore and directly compare the effect of compositional heterogeneity on their dataset at the character state, taxon and partition level. Rather than informing the user that a certain sequence or taxa is compositionally heterogeneous, RCFV allows users to understand whether recoding or masking might be better for their dataset (indicated by skewed csRCFV values), or whether taxon removal might be necessary (indicated by skewed tsRCFV values). In this way, RCFV explores and quantifies variation in character state distribution across a dataset, where other metrics identify and distinguish. In addition, whilst the homogeneity at any given site, as measured by matched-pair tests, is an important component of understanding compositional heterogeneity, the vast majority of currently used models assume homogeneity is a property of the whole alignment [31]. Average relative frequencies (or equilibrium frequencies) are taken from the source alignment to inform the substitution matrix Q, hence the deviation from the average relative frequency is of key importance to understanding compositional heterogeneity in a model that assumes homogeneity. In empirical models such as WAG [5] that do not source their equilibrium frequencies from the source alignment, tsRCFV, csRCFV and RCFV can inform users as to how closely their own alignment reflects these empirical frequencies. In CAT models [32], Q matrices are applied to Dirichlet-selected site categories and hence have their own unique equilibrium frequencies. As sites are classified into a higher number of substantially different categories they are less susceptible to strong deviations from estimated equilibrium frequencies of the whole alignment. However, in these cases, tsRCFV is still informative with regards to compositional heterogeneity between taxa, and csRCFV and RCFV-type metrics can help users understand the dataset CAT is partitioning into categories. Seeing the dataset as your model is liable to see it—through average relative frequencies—and being able to take measures to ameliorate these problems from a shared perspective is important when working with large datasets.

In this study, we assess whether RCFV values are truly independent of the number of taxa and positions in the compared datasets. Using 10,000 simulated datasets, we recorded the variation in RCFV values across 10 taxon categories (ranging from 50 to 500 taxa in 50 taxa steps) and 10 site categories (ranging from 900 to 9000, in steps of 900, for DNA, and from 300 to 3000, in steps of 300, for amino acids). We then expanded our simulations to account for the super-long phylogenetic datasets that are becoming commonplace, accounting for both 100,000 and 500,000 nucleotide and amino acid positions. As RCFV type metrics were shown to be affected by changing sequence length, we additionally explored the effect of missing data. In response to our findings, we introduce a truly normalised Relative Compositional Frequency Variation value family (nRCFV, ncsRCFV, ntsRCFV). These new metrics add a normalisation constant to each of the different RCFV values (total, character-specific, taxon-specific) to mitigate the effect of increasing taxa number and sequence length. Finally, we explore the effect of nRCFV on real data when compared to RCFV by reanalysing the Kocot et al. [14] dataset and find a large effect of sequence length on data selection in empirical data which results in marked topological differences, even when analysed under the same model.

## Methods

To assess the utility of RCFV, 10,000 simulation datasets were generated. Nucleotide simulation datasets were generated under the GTR model [33] using Seq-Gen v1.3.2 [34], and amino acid simulation datasets were generated under the WAG model [5], with an equal rate of evolution applied to all edges, using simSeq in Phangorn 2.1 in R [35]. A uniform distribution of probability of change across sites was also used. The command line used for each program was as follows:

- seq-gen-m GTR-n 100-l $Length-of <$Tree> $Output
- simSeq($Tree, l = $Length, type = “AA”, model = “WAG”)

**Fig. 1:**
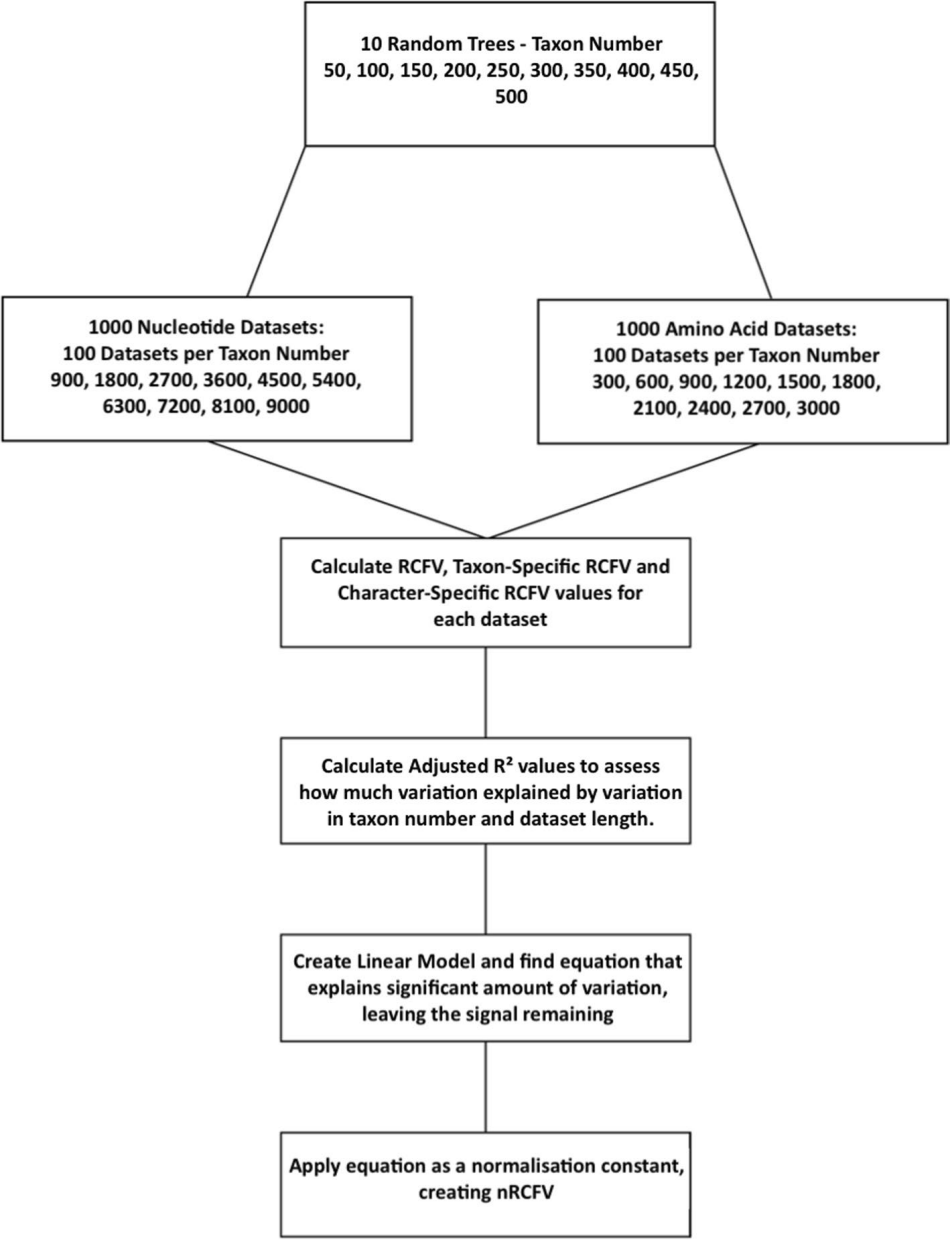
Flow chart showing the analyses conducted in this study to determine the effect of the number of positions and taxa on RCFV, csRCFV and tsRCFV and thereafter the normalization constant of RCFV, csRCFV and tsRCFV

Simulation datasets were calculated in 1000 ‘bins’ of 100 datasets each—taxa at intervals of 50 from 50 taxa to 500 and sequences at intervals of 300 from 300 to 3000 (Fig. 1) positions for amino acids and 900 to 9000 positions for nucleotides. Rtree in ape 5.6-2 was used to create one randomly generated tree for each taxa bin [35, 36], with edge lengths drawn from a continuous uniform distribution, and the same trees were used for each taxa bin at every sequence length interval. These trees are available in our Supplemental Information, hosted at our DataDryad link. As the ability of RCFV to describe compositional heterogeneity was not in question [12], these simulation datasets were created with the intent of being compositionally homogenous, thus allowing any biasing effect due to an increasing number of taxa or positions to be easily identifiable as the only changing variables. The total, taxon-specific and character-specific RCFVs for each dataset were collected using BaCoCa v1.1 [23] under default conditions. Goodness of fit was assessed using linear regression modelling in R 4.1.0 to fit a curve based on both dataset length and taxa number.

To accommodate for the size of modern “super massive” phylogenetic datasets, a further four datasets for both amino acids and nucleotides were simulated using the same methods—50 taxa and 500 taxa at both 100,000 and 500,000 positions. These datasets were then used to assess the consistency of the normalisation constant applied in the following step.

### Normalization of RCFV values

Following the calculation of lines of best fit for the RCFV dataset vs both dataset sequence length and taxon number, a new normalising constant was determined from the line by observing the R^2^ value. This constant was then applied to RCFV to create the new normalised RCFV, henceforth referred to as nRCFV. The same methodology was then applied to the character-specific and taxon-specific RCFV values to create the normalised character-specific and taxon-specific RCFV value (or ncsRCFV and ntsRCFV), which both explained significant amounts of variation in the dataset. This normalization procedure was applied independently to both nucleotide and amino acid datasets as that they are different owing to the change in the number of character states between amino acid and nucleotide datasets (from 20 to 4).

The new normalized RCFV (nRCFV) values can be calculated from the original RCFV values using the following equation:

4 $$nRCFV= \frac{{\varvec{R}}{\varvec{C}}{\varvec{F}}{\varvec{V}}}{{p}^{-0.5}*{n}^{0.01}*c*100}$$

where *p* is the number of positions, *n* is the number of taxa and *c* is the number of character states in the dataset.

The new normalisation constant that was discovered for taxon-specific RCFV is as follows:

5 $$ntsRCFV= \frac{{\varvec{t}}{\varvec{s}}{\varvec{R}}{\varvec{C}}{\varvec{F}}{\varvec{V}}}{{p}^{-0.5}*{n}^{-1}*c*100}$$

The normalization model for character specific RCFV, measuring the relative compositional frequency of individual amino acids and nucleotides as well as combination of these based on specific properties like hydrophilia, polarity, and charge for amino acids and purines/pyrimidines and AT/GC for nucleotides was the same:

6 $$ncsRCFV= \frac{{\varvec{c}}{\varvec{s}}{\varvec{R}}{\varvec{C}}{\varvec{F}}{\varvec{V}}}{{p}^{-0.5}*100}$$

### Exploring the effect of missing data on RCFV

Considering the biasing effect of sequence length and taxon number, we then considered missing data. Using the Kocot et al. [14] dataset for Lophotrochozoa, we simulated 100 datasets using alisim’s alignment mimic option as implemented in IQTree v2.2.0 [37, 38] for each of the 6 missing dataset categories present in that analysis, ranging from 18.17 to 38.43%. As per Kocot et al. [14] methodology, missing data was classified as the presence of ambiguity characters, gaps or a lack of sampling (or absence) of the target gene in the taxon. The alignment mimic takes the properties of the original dataset, including missing data, and constructs a simulation dataset that replicates these conditions. As alisim includes a “no gaps” option, we used this to then remove missing data from each of the 7 categories, creating a further 600 simulation datasets to directly compare the effect of missing data against dataset mimics without missing data. The command used for alisim was as follows:

For “Gapped” datasets: iqtree2 –alisim < Output > -s < Missing Data Dataset > 

For “Ungapped” datasets: iqtree2 –alisim < Output > -s < Missing Data Dataset > –no-copy-gaps.

### Comparing RCFV and nRCFV on real data

We then compared the effect of the normalization constants on real data by taking an example from an existing publication, Kocot et al. [14]. Here, the authors split their dataset into sextiles based on the RCFV value of the individual genes in their multi-locus dataset, producing 10 datasets—one for each sextile specifically, and four compiled datasets of increasing size (the 1st and 2nd sextile, 1st–3rd sextile, 1st–4th sextile and 1st–5th sextile respectively). We first calculated the nRCFV values of each of the 638 genes in the complete dataset, then divided these genes into sextiles in the same manner as the original study, with the 1st sextile comprising 107 genes, the 2nd, 3rd and 6th 106, the 4th 104 and the 5th 109. We then compared the proportion of genes unique to each RCFV/nRCFV sextile pair, and the lengths of each corresponding alignment. For the 1st sextile and the four compiled datasets of increasing size, we reconstructed phylogenies using the new nRCFV-selected genes in IQTree, under the original model conditions (LG + F) used by Kocot et al. [14], and retaining the original multiple sequence alignment. We assessed whether the selection by RCFV or nRCFV influenced the reconstructed topology by calculating the Robinson-Fould (RF) distances of the trees by Kocot et al. [14] based on RCFV values and our new trees based on nRCFV values (Supplemental data on DataDryad) in relation to the tree obtained for all genes (i.e., all six sextiles combined).

## Results

### Evaluating RCFV

**Fig. 2:**
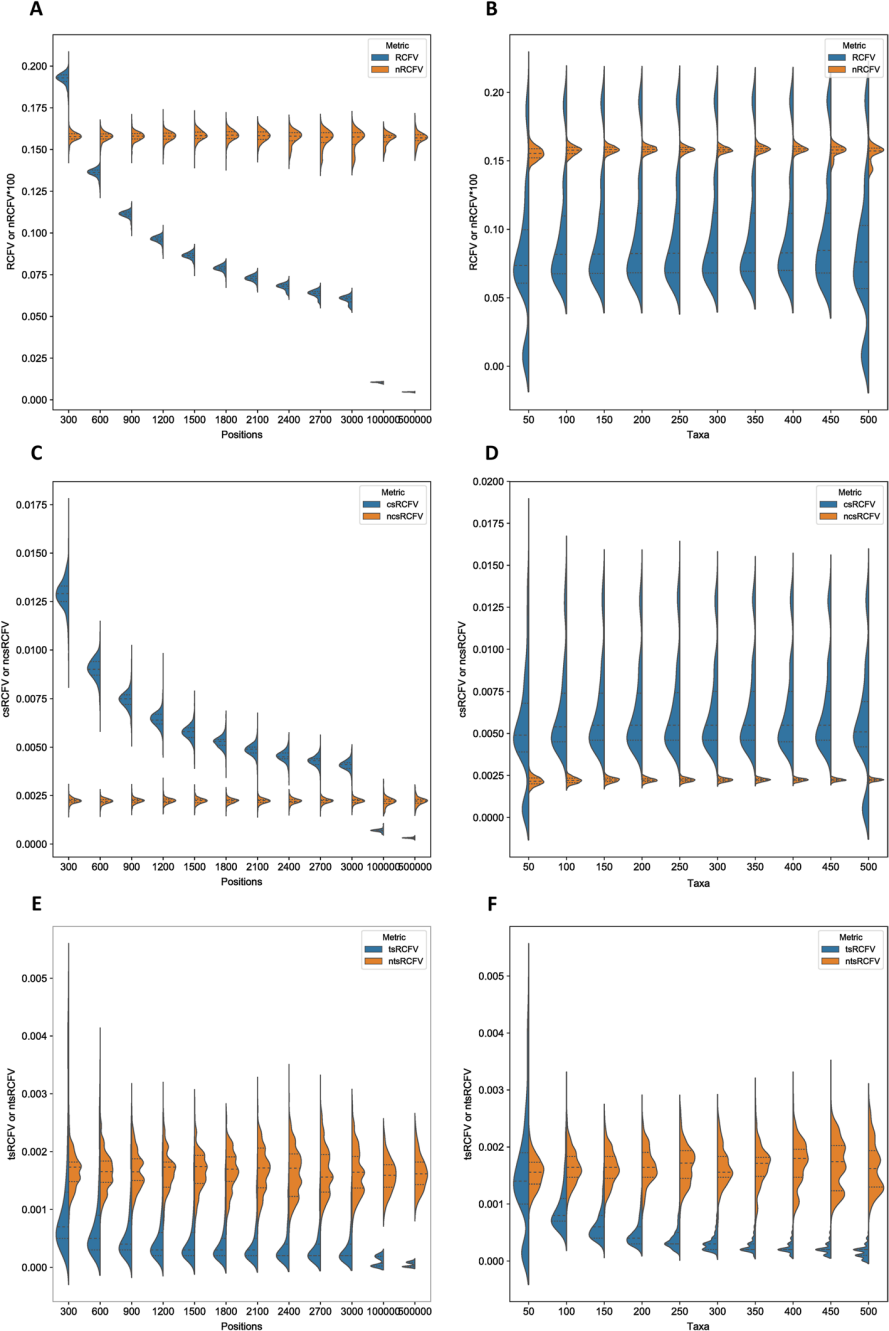
A series of violin plots showing the effect of an increasing number of positions and increasing number of taxa on amino acid data under RCFV, csRCFV, tsRCFV and nRCFV, ncsRCFV and ntsRCFV. Panels **A**, **C** and **E** show the effect of an increasing number of amino acid positions. Panels **B**, **D** and **F** show the effect of an increasing number of taxa

**Fig. 3:**
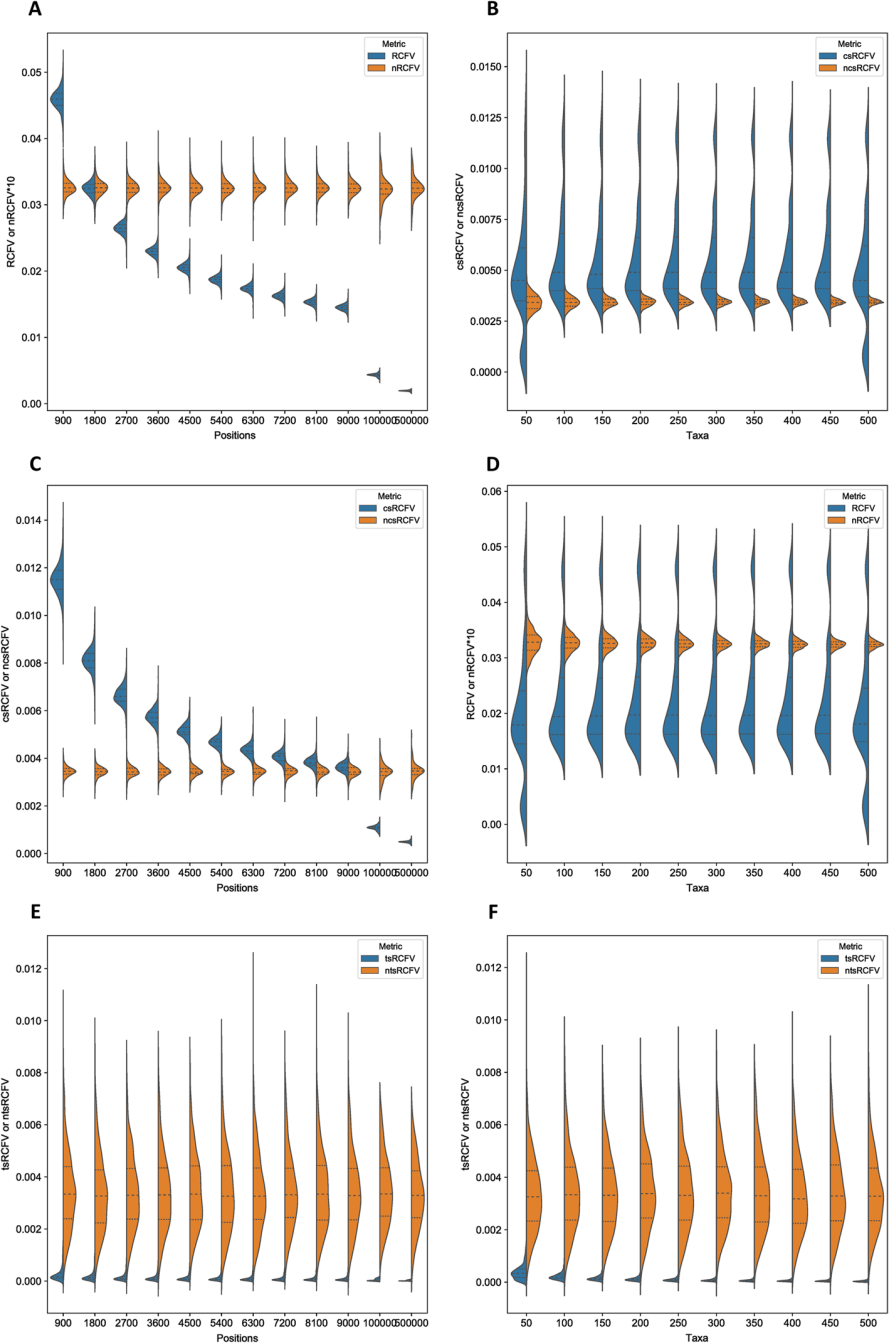
A series of violin plots showing the effect of an increasing number of positions and increasing number of taxa on nucleotide data under RCFV, csRCFV, tsRCFV and nRCFV, ncsRCFV and ntsRCFV. Panels **A**, **C** and **E** show the effect of an increasing number of nucleotide positions. Panels **B**, **D** and **F** show the effect of an increasing number of taxa

Across our 10 × 10 analysis, we found that RCFV was heavily biased by both sequence length and taxa number. As sequence length increases, the RCFV value quickly falls to a plateau, suggesting that at small sequence lengths, RCFV is less capable of distinguishing compositional heterogeneity, and that RCFV becomes more comparable between datasets consisting of longer sequence lengths (Figs. 2A and 3A). In the case of increasing sequence length, this was found to be significantly related (ANOVA *p*-value =  < 2e^−16^) to decreasing RCFV. The same can be shown for nucleotide data, csRCFV and tsRCFV values, but it is less pronounced for csRCFV (Figs. 2C, E and 3C, E, ANOVA *p*-value =  < 2e^−16^). This is a cause of some concern, as it suggests that the statistical power of RCFV is lower with less data and that longer genes are favoured over smaller ones.

In the case of taxa number, the trend is a little bit more complicated for RCFV values. We found that the proportional decrease was instead in variation across a higher and a lower data point, with decreasing variation being found at higher numbers of taxa (Figs. 2B and 3B, ANOVA *p*-value = 0.0001807), though the mean value of RCFV remained relatively stable across taxa number. A similar, but non-significant pattern can be found for the csRCFV values (Figs. 2C and 3C, ANOVA *p*-value = 0.0945). The tsRCFV values display a far less pronounced effect when comparing an increasing number of positions to an increasing number of taxa (Figs. 2E and 3E, ANOVA *p*-value = 0.0005761). With an increasing number of taxa, tsRCFV values decrease. Again, the results for the nucleotide datasets mirror the amino acid results, with higher significance for RCFV and tsRCFV and non-significance for csRCFV (Fig. 2B, D, E respectively, RCFV ANOVA *p*-value =  < 2e^−16^, csRCFV ANOVA *p*-value = 0.3078, tsRCFV ANOVA =  < 2.2e^−16^).

### Normalization of the RCFV values

As the RCFV value was shown to be biased with regards to both taxon number and sequence length, we then resolved to discover and implement the theoretical normalisation constant that would ameliorate the effect of these factors on RCFV. The normalisation procedure with respect to the number of positions and taxa resulted in different normalisation formulae for the RCFV values for the complete dataset, as well as the character- and taxon-specific values.

This new nRCFV was then cross-referenced against the original dataset to ensure that both dataset size factors no longer biased the value. This was established first by observing the trendline of the data. In both cases, there is no longer an increase or decrease of the values with respect to the number of positions and taxa (Figs. 2C–F and 3C–F). By adjusting for taxa, the splitting into two optima, one at a higher and one at a lower value, is no longer observable (Fig. 2D). By multiplying the number of character states by 100, nRCFV produces values that are comparable with the difference between the character state frequencies and mean character state frequencies present in the dataset, increasing usability. Then, we assessed the residuals within our predicted linear model to ensure that the new nRCFV value explained a significant amount of dependent variation within the dataset (Adjusted R^2^ = 0.9988). Accordingly, the remaining minor variation could be explained by dataset-to-dataset differences independent of the above dataset size factors.

Our linear model for taxon-specific RCFV returned an adjusted R^2^ value of 0.9376. Plotting the new normalized taxon-specific RCFV values against either number of positions or taxa (Figs. 2E, F and 3E, F) revealed change similar to that of nRCFV values for amino acid datasets (Fig. 2C, D). Moreover, ntsRCFV values increased substantially more than nRCFV did for higher values. This results in ntsRCFV values up to a 100-fold larger than the original tsRCFV, which allows them to be reasonably compared with total nRCFV (Figs. 2E, F and 3E, F).

This normalised taxon-specific RCFV had the lowest adjusted R^2^ of all normalizations at 0.9376 on our combined nucleotide and amino acid dataset. We explain this anomalous variation in the final value through the ability of tsRCFV to explain more real variation in the dataset without being normalised. The non-normalised tsRCFV returned an R^2^ value of 0.6726 and 0.5263 when compared against variation in taxa and 0.1520 and 0.1108 when compared against positions (Figs. 2E, F and 3E, F). For the number of positions, this is far below the other RCFV values, where over 97% of the variation could be explained by their variation (Fig. 2A, C, E). The number of taxa explains more of the variation in tsRCFV (Figs. 2E, F and 3E, F), but tsRCFV values do not show the distribution around two optima as the other RCFV values do (Figs. 2B, D, F and 3B, D, F). Unnormalised taxon-specific RCFV shows an adjusted R^2^ value of 0.5743 on amino acid data and 0.354 on nucleotide data when comparing against a simple model of Positions + Taxa, suggesting that variation in these values already explains less of the variation in RCFV (see Supplemental Information on Datadryad for raw simulation data and linear model results). In addition, additional parameters could not be added to the normalization model without decreasing the adjusted R^2^ relative to the unadjusted R^2^, suggesting overfitting.

The ncsRCFV value is not biased by the number of taxa *n*, although total RCFV and tsRCFV are. This agrees with the extremely low R^2^ values (< 0.001, Figs. 2D and 3D) and that the ANOVA results were non-significant for taxa. Our model for single amino acid character states returned an adjusted R^2^ of 0.9758, and for single nucleotides, the adjusted R^2^ of ncsRCFV is 0.9774. Additionally, as with ntsRCFV, the proportional increase of the ncsRCFV in comparison to the csRCFV is far more prominent than in nRCFV values. This allows for comparison versus whole dataset nRCFV directly, rather than as a proportion of this total value.

### Is RCFV affected by the number of positions, taxa and possible character states?

As mentioned in the Introduction, in theory the RCFV should be independent of the number of positions and taxa as the frequency is used and a normalization on the number occurs for individual values for each character state and taxon. However, the results herein showed that this is not the case. This raises the question of whether the principal assumption was wrong. To answer this, we can perform a simple thought experiment.

Let dataset I be a dataset with only two character-states—purines (R) and pyrimidines (Y)—*n* taxa and *p* positions. Then, the RCFV_I_ can be calculated using the number of occurrences (O) of each character state per taxon:

7 $${RCFV}_{I}=\sum_{i=1}^{n}\frac{\left|{\mu }_{Ri}-{\overline{\mu }}_{R}\right|+\left|{\mu }_{Yi}-{\overline{\mu }}_{Y}\right|}{n}$$

with

$${\mu }_{Ri}=\frac{{O}_{Ri}}{p}$$

$${\overline{\mu }}_{R}=\frac{\sum_{i=1}^{n}{O}_{Ri}/p}{n}$$

8 $$\left|{\mu }_{Ri}-{\overline{\mu }}_{R}\right|=\left|\frac{{O}_{Ri}}{p}-\frac{\sum_{i=1}^{n}\frac{{O}_{Ri}}{p}}{n}\right|=\left|\frac{{O}_{Ri}}{p}-\frac{\sum_{i=1}^{n}{O}_{Ri}}{np}\right|=\left|\frac{n}{n}*\frac{{O}_{Ri}}{p}-\frac{\sum_{i=1}^{n}{O}_{Ri}}{np}\right|=\frac{1}{np}\left|n{O}_{Ri}-\sum_{i=1}^{n}{O}_{Ri}\right|$$

The same applies to Y. Hence,

9 $${RCFV}_{I}=\sum_{i=1}^{n}\frac{\left|n*{O}_{Ri}-\sum_{i=1}^{n}{O}_{Ri}\right|+\left|n*{O}_{Yi}-\sum_{i=1}^{n}{O}_{Yi}\right|}{{n}^{2}*p}$$

Consider dataset II, which is generated by copying each position in dataset I exactly once, so that we have *n* taxa and 2*p positions with each character state R and Y occurring exactly twice as often in each taxon. Then, RCFV_II_ can be calculated using the number of occurrences (2*O) of each character state per taxon using eq. #7. As before we calculate:

$${\mu }_{Ri}=\frac{{2*O}_{Ri}}{2*p}=\frac{{O}_{Ri}}{p}$$

$${\overline{\mu }}_{R}=\frac{\sum_{i=1}^{n}{(2*O}_{Ri})/(2*p)}{n}=\frac{\sum_{i=1}^{n}{O}_{Ri}/p}{n}$$

This resolves to the exact same as Eq. 8. The same applies to Y again. Accordingly, we also get the same as in Eq. 9 for RCFV_II_.

Finally, consider dataset III, which is generated by copying each taxon in dataset I exactly once, so that we have 2*n taxa and *p* positions with each character state R and Y occurring exactly twice as often at each position. The number of occurrences (O) per taxon remains unchanged for each character state. Then, the RCFV can be calculated the following way using:

10 $${RCFV}_{III}=\sum_{i=1}^{2n}\frac{\left|{\mu }_{Ri}-{\overline{\mu }}_{R}\right|+\left|{\mu }_{Yi}-{\overline{\mu }}_{Y}\right|}{2*n}$$

As before we calculate:

$${\mu }_{Ri}=\frac{{O}_{Ri}}{p}$$

$${\overline{\mu }}_{R}=\frac{\sum_{i=1}^{2n}{O}_{Ri}/p}{2n}=\frac{2*\sum_{i=1}^{n}{O}_{Ri}/p}{2n}=\frac{\sum_{i=1}^{n}{O}_{Ri}/p}{n}$$

The same applies to Y and we can use Eq. 8 in Eq. 10:

$${RCFV}_{III}=\sum_{i=1}^{2n}\frac{\left|n*{O}_{Ri}-\sum_{i=1}^{n}{O}_{Ri}\right|+\left|n*{O}_{Yi}-\sum_{i=1}^{n}{O}_{Yi}\right|}{{2*n}^{2}*p}=\frac{1}{2}\sum_{i=1}^{2n}\frac{\left|n*{O}_{Ri}-\sum_{i=1}^{n}{O}_{Ri}\right|+\left|n*{O}_{Yi}-\sum_{i=1}^{n}{O}_{Yi}\right|}{{n}^{2}*p}=\frac{1}{2}\left(2*\sum_{i=1}^{n}\frac{\left|n*{O}_{Ri}-\sum_{i=1}^{n}{O}_{Ri}\right|+\left|n*{O}_{Yi}-\sum_{i=1}^{n}{O}_{Yi}\right|}{{n}^{2}*p}\right)=\sum_{i=1}^{n}\frac{\left|n*{O}_{Ri}-\sum_{i=1}^{n}{O}_{Ri}\right|+\left|n*{O}_{Yi}-\sum_{i=1}^{n}{O}_{Yi}\right|}{{n}^{2}*p}$$

This equation is the same as Eq. 9.

In both cases of exact duplications of either position or taxa, the same RCFV will be calculated. Hence, the theoretical assumption that the RCFV values should be taxon- and position-independent is, in principle, correct. However, the simulation data showed a clear dependence on both the number of positions and taxa. A possible explanation for this could be that the size of the dataset has an effect on the calculation of the data. As the dataset upon which RCFV is calculated becomes larger in both breadth and length, a single compositional change affects the RCFV value across the entire dataset less. This is because each change then represents a smaller proportion of the entire RCFV value, which is totalled across all positions and taxa. These observations were corroborated by our analysis of both character and taxon-specific RCFV values, as the former only needed adjustment for the number of positions, not the number of taxa, which is likely due to the reduction in variance caused by measuring only single data points.

### The effect of size on RCFV

This effect of size can be shown in a simple thought experiment. Consider dataset IV with four character states (e.g., A,C,T,G), *n* taxa and *p* positions from which we will generate a new dataset V by changing one character state (A) to another (G) in just one taxon (N). For dataset IV, the RCFV_IV_ can be calculated by separating one taxon from the others, bearing in mind that the RCFV values for C and T (together RCFV_Y_) are not affected by this change:

11 $${RCFV}_{IV}=\sum_{i=1}^{n}\frac{\left|{\mu }_{Ai}-{\overline{\mu }}_{A}\right|+\left|{\mu }_{Gi}-{\overline{\mu }}_{G}\right|+\left|{\mu }_{Ci}-{\overline{\mu }}_{C}\right|+\left|{\mu }_{Ti}-{\overline{\mu }}_{T}\right|}{n}= \sum_{i=1}^{n}\frac{\left|{\mu }_{Ai}-{\overline{\mu }}_{A}\right|}{n}+\sum_{i=1}^{n}\frac{\left|{\mu }_{Gi}-{\overline{\mu }}_{G}\right|}{n}+\sum_{i=1}^{n}\frac{\left|{\mu }_{Ci}-{\overline{\mu }}_{C}\right|}{n}+\sum_{i=1}^{n}\frac{\left|{\mu }_{Ti}-{\overline{\mu }}_{T}\right|}{n}=\sum_{i=1}^{n}\frac{\left|{\mu }_{Ai}-{\overline{\mu }}_{A}\right|}{n}+\sum_{i=1}^{n}\frac{\left|{\mu }_{Gi}-{\overline{\mu }}_{G}\right|}{n}+{RCFV}_{Y}=\left(\left(\sum_{i=1}^{n-1}\frac{\left|{\mu }_{Ai}-{\overline{\mu }}_{A}\right|}{n}\right)+\frac{\left|{\mu }_{AN}-{\overline{\mu }}_{A}\right|}{n}\right)+\left(\left(\sum_{i=1}^{n-1}\frac{\left|{\mu }_{Gi}-{\overline{\mu }}_{G}\right|}{n}\right)+\frac{\left|{\mu }_{GN}-{\overline{\mu }}_{G}\right|}{n}\right)+{RCFV}_{Y}$$

Consider now dataset V with one character state (A) changing to another (G) in just one taxon (N), but retaining *n* taxa and *p* positions. Denoting the changed frequencies for A and G with a star we can apply Eq. 11 here:

12 $${RCFV}_{V}=\left(\left(\sum_{i=1}^{n-1}\frac{\left|{\mu }_{Ai}-{\overline{\mu }}_{A}^{*}\right|}{n}\right)+\frac{\left|{\mu }_{AN}^{*}-{\overline{\mu }}_{A}^{*}\right|}{n}\right)+\left(\left(\sum_{i=1}^{n-1}\frac{\left|{\mu }_{Gi}-{\overline{\mu }}_{G}^{*}\right|}{n}\right)+\frac{\left|{\mu }_{GN}^{*}-{\overline{\mu }}_{G}^{*}\right|}{n}\right)+{RCFV}_{Y}$$

With

$${\mu }_{AN}^{*}=\frac{{O}_{AN}+1}{p}={\mu }_{AN}+\frac{1}{p}$$

$${\mu }_{GN}^{*}=\frac{{O}_{GN}-1}{p}={\mu }_{GN}-\frac{1}{p}$$

$${\overline{\mu }}_{A}^{*}=\frac{\left(\sum_{i=1}^{n-1}\frac{{O}_{Ai}}{p}\right)+\frac{{O}_{AN}+1}{p}}{n}=\frac{\left(\sum_{i=1}^{n-1}\frac{{O}_{Ai}}{p}\right)+\frac{{O}_{AN}}{p}+\frac{1}{p}}{n}=\frac{\left(\sum_{i=1}^{n}\frac{{O}_{Ai}}{p}\right)+\frac{1}{p}}{n}={\overline{\mu }}_{A}+\frac{1}{np}$$

$${\overline{\mu }}_{G}^{*}=\frac{\left(\sum_{i=1}^{n-1}\frac{{O}_{Gi}}{p}\right)+\frac{{O}_{GN}-1}{p}}{n}=\frac{\left(\sum_{i=1}^{n-1}\frac{{O}_{Gi}}{p}\right)+\frac{{O}_{GN}}{p}-\frac{1}{p}}{n}=\frac{\left(\sum_{i=1}^{n}\frac{{O}_{Gi}}{p}\right)-\frac{1}{p}}{n}={\overline{\mu }}_{G}-\frac{1}{np}$$

Equation 12 can be transformed to:

13 $${RCFV}_{V}=\left(\left(\sum_{i=1}^{n-1}\frac{\left|{\mu }_{Ai}-\left({\overline{\mu }}_{A}+\frac{1}{np}\right)\right|}{n}\right)+\frac{\left|{\mu }_{AN}+\frac{1}{p}-\left({\overline{\mu }}_{A}+\frac{1}{np}\right)\right|}{n}\right)+\left(\left(\sum_{i=1}^{n-1}\frac{\left|{\mu }_{Gi}-\left({\overline{\mu }}_{G}-\frac{1}{np}\right)\right|}{n}\right)+\frac{\left|{\mu }_{GN}-\frac{1}{p}-\left({\overline{\mu }}_{G}-\frac{1}{np}\right)\right|}{n}\right)+{RCFV}_{Y}=\left(\left(\sum_{i=1}^{n-1}\frac{\left|{\mu }_{Ai}-{\overline{\mu }}_{A}-\frac{1}{np}\right|}{n}\right)+\frac{\left|{\mu }_{AN}-{\overline{\mu }}_{A}+\frac{n-1}{np}\right|}{n}\right)+\left(\left(\sum_{i=1}^{n-1}\frac{\left|{\mu }_{Gi}-{\overline{\mu }}_{G}+\frac{1}{np}\right|}{n}\right)+\frac{\left|{\mu }_{GN}-{\overline{\mu }}_{G}-\frac{n-1}{np}\right|}{n}\right)+{RCFV}_{Y}$$

**Fig. 4:**
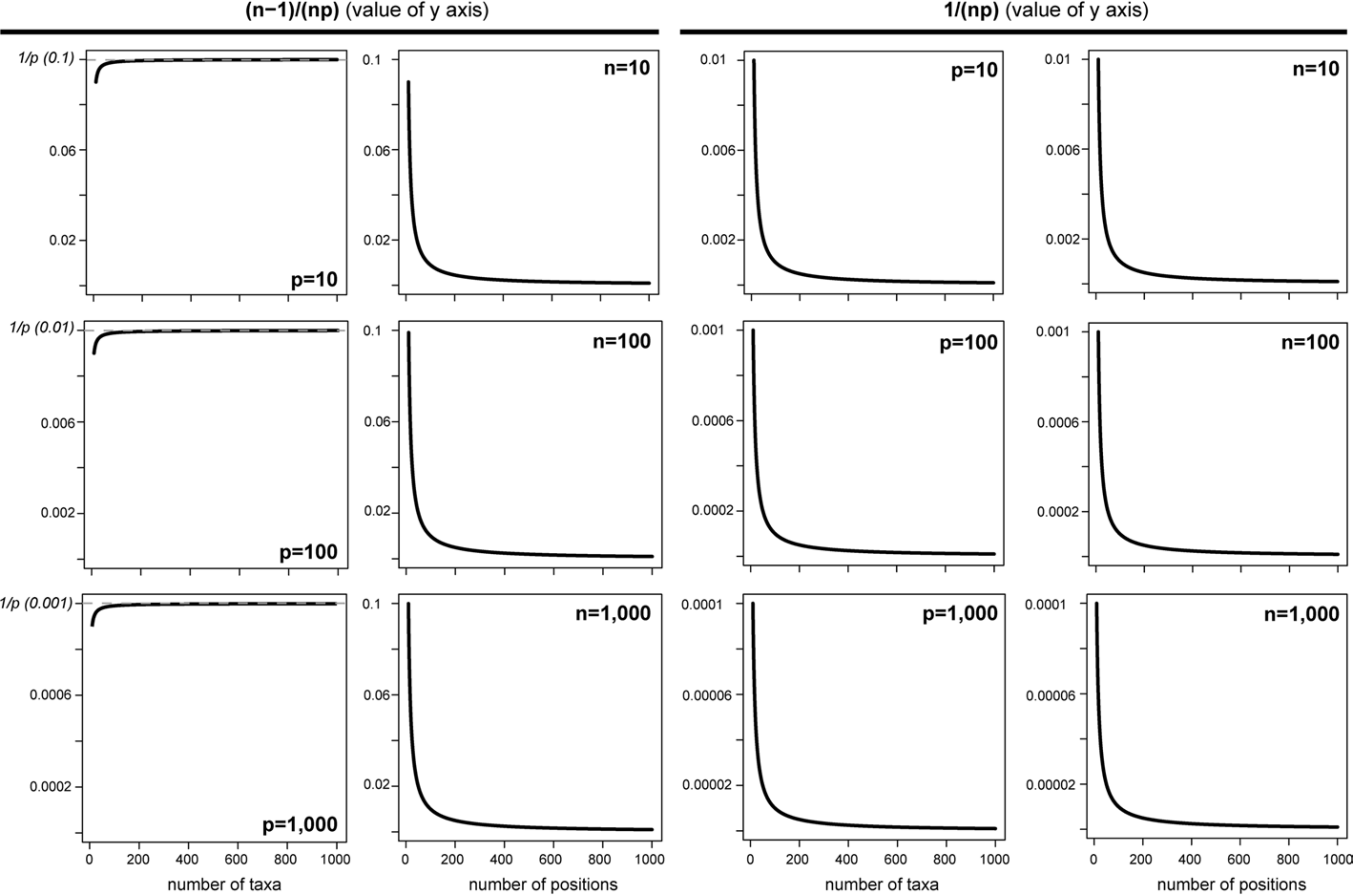
Plotting of the effect of the number of positions or taxa on the two factors (i.e., (n − 1)/(np) and 1/(np)) obtained in Eq. 11 given different constant values for the number of positions (p) or taxa (t). The upper limit at 1/p for the number of taxa for the factor (n − 1)/(np) is indicated with a dashed line

Comparing Eq. 13 with 11 shows that the values of the affected taxon and character states are changed by a factor *(n-1)/(np)*. Hence, RCFV values clearly depend on the number of positions and taxa. Moreover, the lower both values are, the stronger the effect. When *n* approaches ∞, *(n-1)≈n* and *(n-1)/n* approaches 1 asymptotically from lower values. Accordingly, the factor becomes *1/p*. Hence, at high *n* only *p* has an effect on the RCFV value. The effect on the affected taxon increases with increasing number of taxa, capped by the number of positions (i.e., *1/p*). On the other hand, as *p* approaches ∞, the factor asymptotically approaches 0 independent of *n*. Hence, the effect of the number of positions is negligible at high numbers of positions. Figure 4 provides an example of both behaviours.

However, the unaffected taxa also change due to the change in average frequencies across all taxa. The factor *1/(np)* is smaller than for the affected taxon, but both number of taxa and positions have an effect. However, in this case, when either *n* or *p* approaches ∞, the factor asymptotically approaches 0. Hence, in this case, both become negligible at higher numbers of positions, taxa, or both (Figs. 2, 3 and 4). Additionally, even though the factor is much smaller for each individual taxon than for the affected taxon, the effect is summed up over *n-1* taxa and hence can be substantial. The simulated data in general were also sensitive to number of taxa and asymptotically approached lower values with increasing number of taxa, indicating a decreasing influence on the RCFV calculation. Hence, the decreasing influence of the unaffected taxa cumulatively seems to outweigh the increasing, but capped, influence on the affected taxon. Moreover, the contradictory influence of the number of taxa on the affected and unaffected taxa with an increasing number of taxa can also be seen in the simulated data. The RCFV and csRCFV converge on two optimal values, a high one and a low one (Figs. 2, 3 and 4). Finally, as the factor is part of a summation of absolute values in both the affected taxon and the other taxon, the effects will not cancel each other out.

### The effect of the number of possible character states on RCFV

Finally, the number of possible character states is also relevant to how strongly both factors *(n-1)/(np)* and *1/(np)* can influence the calculation of the total RCFV value. In the example above with four possible character states, only two out of four are affected by the change, while the other two are not affected. Hence, only 50% of the RCFV calculation is affected by the change. In contrast, if we take the other example from above with only two character states and assuming, for example, a single change from Y to R, Eq. 13 would be changed to:

14 $$RCFV=\left(\left(\sum_{i=1}^{n-1}\frac{\left|{\mu }_{Ri}-{\overline{\mu }}_{R}-\frac{1}{np}\right|}{n}\right)+\frac{\left|{\mu }_{RN}-{\overline{\mu }}_{R}+\frac{n-1}{np}\right|}{n}\right)+\left(\left(\sum_{i=1}^{n-1}\frac{\left|{\mu }_{Yi}-{\overline{\mu }}_{Y}+\frac{1}{np}\right|}{n}\right)+\frac{\left|{\mu }_{YN}-{\overline{\mu }}_{Y}-\frac{n-1}{np}\right|}{n}\right)$$

Hence, both character states are affected by these factors and 100% of the RCFV calculation. In contrast, if one considers 20 possible character states like amino acids and assuming, for example, a change from glycine (G) to alanine (A), the Eq. 13 would be changed to:

15 $$RCFV=\left(\left(\sum_{i=1}^{n-1}\frac{\left|{\mu }_{Ai}-{\overline{\mu }}_{A}-\frac{1}{np}\right|}{n}\right)+\frac{\left|{\mu }_{AN}-{\overline{\mu }}_{A}+\frac{n-1}{np}\right|}{n}\right)+\left(\left(\sum_{i=1}^{n-1}\frac{\left|{\mu }_{Gi}-{\overline{\mu }}_{G}+\frac{1}{np}\right|}{n}\right)+\frac{\left|{\mu }_{GN}-{\overline{\mu }}_{G}-\frac{n-1}{np}\right|}{n}\right)+{RCFV}_{sum of all other 18}$$

Accordingly, only two of the 20 character-state calculations are affected by the factor and hence only 10% of the RCFV calculation. The overall effect on the value will still be present, but at a much smaller level. Hence, not only the number of positions and taxa are relevant, but also the number of character-states, as already concluded above. Accordingly, the normalization has to be adjusted for each possible number of character states individually. The same normalization cannot be applied, for example, for values considering all nucleotides or all amino acids. It must be independently determined. A consequence of this is that without normalization one can compare only RCFV values which are determined using the same number of character states. However, it is important to note that just the number of character states is important here, not their nature. For example, binary states can be compared directly independently of whether they are, for example, absence/presence coding or RY coding. This has also been shown above by using the same model for all one character-state calculations.

Character- and taxon-specific RCFV are affected by the number of taxa and positions in a similar way. This can be seen by considering the corresponding parts of the RCFV_V_ calculation by transforming Eq. 13 using Eqs. 2 and 3, respectively:

$$cs{RCFV}_{V-A}=\left(\left(\sum_{i=1}^{n-1}\frac{\left|{\mu }_{Ai}-{\overline{\mu }}_{A}-\frac{1}{np}\right|}{n}\right)+\frac{\left|{\mu }_{AN}-{\overline{\mu }}_{A}+\frac{n-1}{np}\right|}{n}\right)$$

$${csRCFV}_{V-G}=\left(\left(\sum_{i=1}^{n-1}\frac{\left|{\mu }_{Gi}-{\overline{\mu }}_{G}+\frac{1}{np}\right|}{n}\right)+\frac{\left|{\mu }_{GN}-{\overline{\mu }}_{G}-\frac{n-1}{np}\right|}{n}\right)$$

$${tsRCFV}_{V-N}=\frac{\left|{\mu }_{AN}-{\overline{\mu }}_{A}+\frac{n-1}{np}\right|}{n}+\frac{\left|{\mu }_{GN}-{\overline{\mu }}_{G}-\frac{n-1}{np}\right|}{n}+{tsRCFV}_{Y}$$

16 $${tsRCFV}_{V-others}=\frac{\left|{\mu }_{Ai}-{\overline{\mu }}_{A}-\frac{1}{np}\right|}{n}+\frac{\left|{\mu }_{Gi}-{\overline{\mu }}_{G}+\frac{1}{np}\right|}{n}+{tsRCFV}_{Y}$$

### Comparison of RCFV and nRCFV values in empirical data

**Fig. 5:**
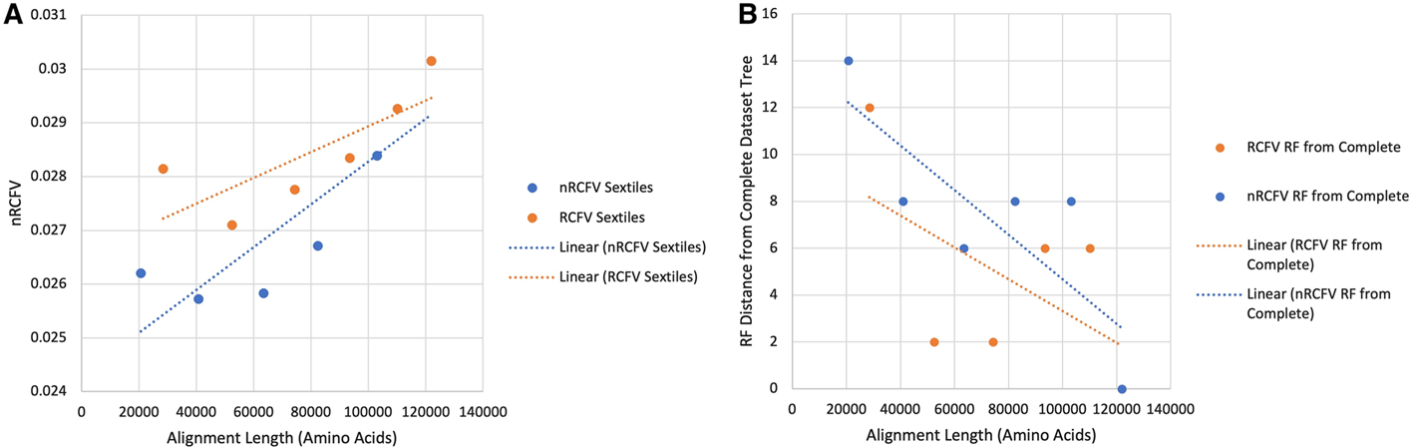
The effect of nRCFV and RCFV on gene selection resulting in different topologies. Panel **A** shows the increasing RCFV and nRCFV values (in Orange and Blue, respectively). Panel **B** shows the RF distance of the increasing sextile datasets selected by RCFV (Orange) and nRCFV (Blue) compared to a “complete” dataset containg all genes used in the original Kocot et al. [14] study

The analyses of the different sextiles by Kocot et al. [14] based on compositional heterogeneity revealed substantial differences in the assignment of the genes to different sextiles depending on the normalization of RCFV (Table 1). We found that, in the case of the 1st sextile, 44% of the genes selected by nRCFV were not selected by RCFV for the same sextile. Of these 47 genes, 30 belonged to the 2nd sextile, 15 to the 3rd and 1 each to the 4th and 5th. In addition, the alignment length of the dataset under RCFV was 28,490, compared to 20,672 in nRCFV (Table 1, Fig. 5A). Similarly, the sextile containing only the highest nRCFV values did not share 62% of its sequences with the same RCFV sextile. The alignment length increased from 11,813 based on this RCFV sextile to 18,887 based on nRCFV. The percentage of unshared genes is even more notable in the middle sextiles ranging from 72% in the 4th to 81% in the 5th (Table 1). Here sequences placed in a particular sextile by nRCFV are similarly likely to be found in the neighbouring RCFV sextiles.

**Table 1:** Differences in gene selection based on RCFV and nRCFV values given the Kocot et al. [14] dataset

Sextile	Percentage non-shared genes (%)	Alignment length based on RCFV	Alignment length based on nRCFV
1st	44	28,490	20,672
2nd	78	24,037	20,194
3rd	74	21,771	22,596
4th	72	19,211	18,921
5th	81	16,658	20,712
6th	62	11,813	18,887

When the RF distances from the tree based on the complete dataset are assessed, the best sextile selected by RCFV produces a tree that is two steps closer to the complete tree than the best sextile selected by nRCFV (Fig. 5B). As additional sextiles are added to the dataset and more genes shared between the two comparative datasets, the distance to the complete tree decreases, as might be expected. However, this decrease occurs far faster and far more completely in the RCFV selected sextiles, with the second and third sextiles possessing a RF distance of 2 from the complete tree, and the fourth and fifth possessing a RF distance of 6. Whilst a similar pattern is observable in the genes selected by nRCFV, the decline is not nearly as steep nor as noticeable, with the third sextile dataset being the closest to the complete tree at 6, and the fourth and fifth sextiles being recovered at a RF distance of 8 from the complete tree.

With the addition of more heterogeneous sextiles, the nRCFV values increased linearly independently of whether the genes were selected based on RCFV or nRCFV values. However, the nRCFV value was always smaller for genes selected by nRFCV values than for those based on RCFV values, even when only the 6th sextile was excluded from the complete dataset (Fig. 5A).

### RCFV and missing data

In theory, as the number of positions affects the RCFV, then we might expect the variation in the number of positions internally within the dataset to also affect RCFV, as a change at one site in a sequence with more missing sites will be worth proportionally more than a change in a sequence with fewer missing sites. To accomplish this, we took the real Kocot et al. [14] dataset and examined the Missing Data sextiles as per their original analysis. These six datasets range from 18 to 38% missing data, and we then used AliSim [37, 38] to create 100 simulation datasets that mimicked each of these using the alignment mimic command. We then created artificially gapless simulations of these alignments, again using AliSim’s alignment mimic, using the “no gaps” option. In theory, as “no gaps” replaces the missing data with data that is consistent with the overall amino acid proportions of the source alignment, the RCFV value should remain consistent between datasets with missing data and those without if RCFV is not affected by missing data.

**Fig. 6:**
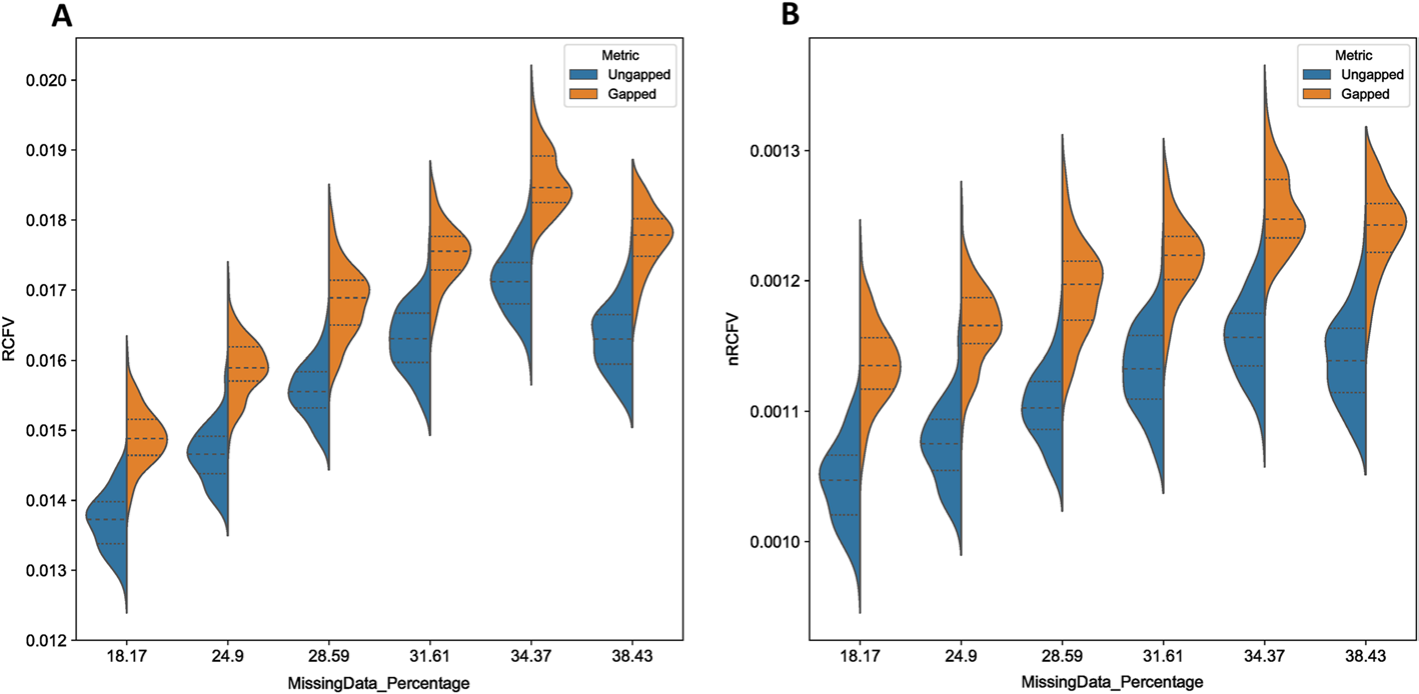
Two violin plots displaying the effect of missing data on RCFV and nRCFV. Panel **A** shows RCFV and Panel **B** nRCFV. Boxes in orange represent the simulation datasets generated by AliSim with missing data and boxes in blue represent the simulation datasets generated by AliSim without missing data. Each category is defined by the percentage missing data of the source dataset, which is present in the orange simulation datasets

**Fig. 7:**
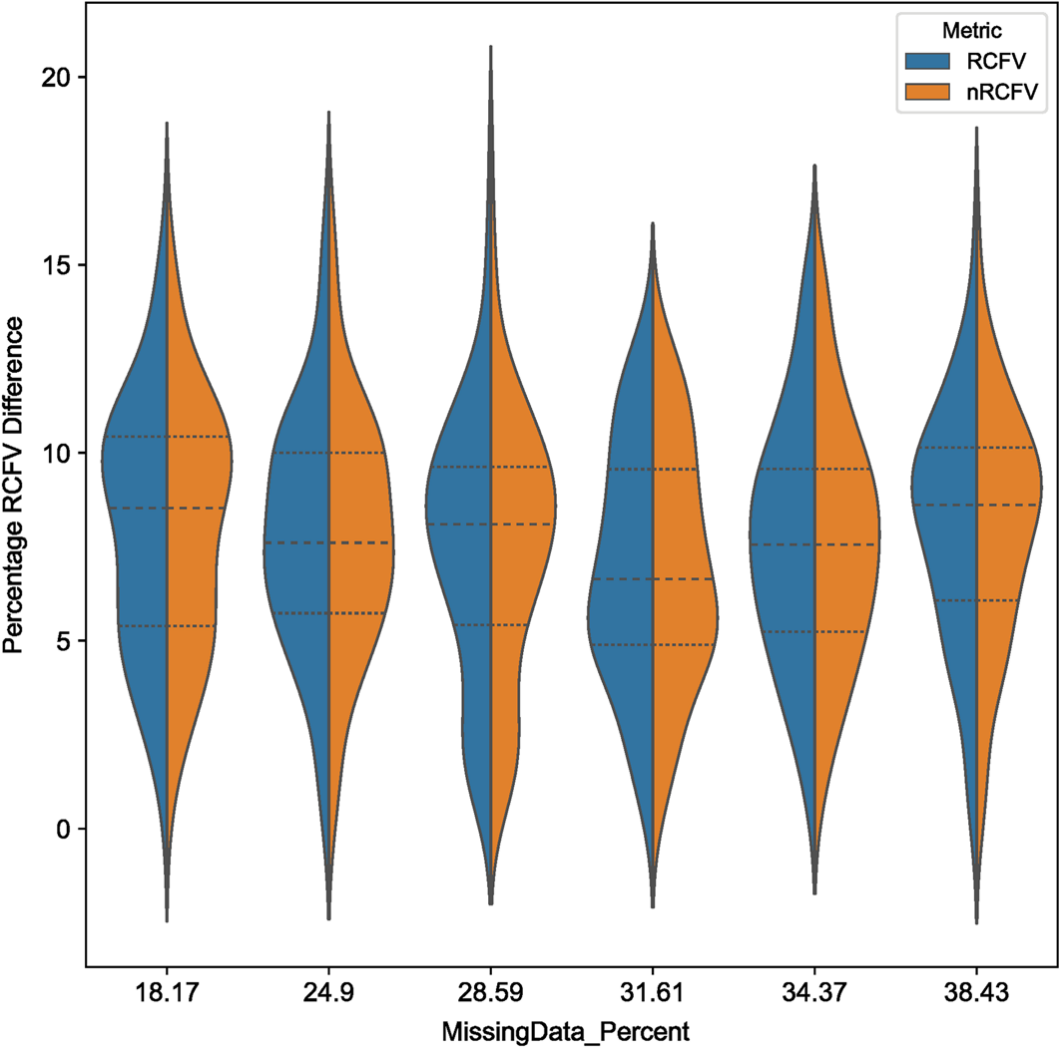
A violin plot showing the effect of missing data on nRCFV and RCFV, expressed as the percentage difference in the metric between the gapped and ungapped datasets. RCFV is expressed in blue and nRCFV in orange

We found that RCFV does appear to increase slightly with an increasing degree of missing data (Fig. 6). However, this effect seems negligible—across all 6 missing data categories, both RCFV and nRCFV the completed alignments show a consistent average increase of 7–8.1% with respect to their missing data pair across the 100 data points. This value is seemingly unaffected by the proportion of missing data in the dataset: the two largest average increases (8.1% and 8%) were observed in both the 18.17% missing data dataset and the 38.43% missing data dataset respectively, whilst the smallest percentage increase was found in the 31.61% missing data dataset. The absolute maximum difference observed was a change of 18.006% found in the 28.59% missing data dataset. This was one of 9 simulations across the dataset that showed more than 15% change: 0.015% of the 600 simulation datasets. Notably, these outlier results were more frequently found in datasets with less missing data: 3 were present in the 24.9% missing data simulations and 3 in the 28.59% missing data simulations, with the remaining 3 being distributed 1 each in 18.17%, 34.37% and 38.43% respectively (Fig. 7). In addition, though AliSim’s alignment mimic option does intend to produce an exact replica of the target dataset, though gapless, we observed small shifts in the amino acid proportions between the original missing data datasets and the gapless simulation datasets, between 0.06% (Simulation Average Freq (L) vs Original Data Freq (L) 0.0958 vs 0.0957) and 7.98% (Simulation Average Freq (H) vs Original Data Freq (H) 0.0220 vs 0.0238), with an average absolute frequency change of 2.68% in the 18.17% dataset. This suggests that the increase in nRCFV and RCFV values may be real and responsive to small changes in compositional homogeneity between the two datasets (See Supplemental Information on Datadryad).

## Discussion

### Bias within RCFV

Our results show that the calculation of the RCFV value is clearly affected by the number of positions and taxa considering both the actual RCFV and the character- and taxon-specific ones. However, we also showed that these effects can be normalized using different models for the RCFV, character- and taxon-specific RCFV values. The inclusion of a correction for the number of character states for the normalization of RCFV and tsRCFV also shows that not only do the number of taxa and position influence the RCFV calculation, but also the number of possible character states. On the other hand, the model for csRCFV is the same; independent of whether single amino acids, single nucleotides or nucleotide combinations (such as pyramidines or purines) are calculated. The reason for this is that in the case of csRCFV only a single character state is considered, even for combinations of amino acids and nucleotides, as they are added together in one frequency value. Hence, the normalization of all forms of RCFV values (i.e., RCFV, csRCFV and tsRCFV) depends on the number of possible character states, not on the nature of the character. For example, a dataset consisting of only four morphological character states or Dayhoff recodings could be normalized using the models for nucleotides. Another consequence of the normalisation procedure is that the new nRCFV value is no longer a summation of the character-specific or taxon-specific RCFV values for each character state or taxon, respectively. Rather, the total nRCFV of the dataset now represents an average value, around which standard deviations can be calculated to determine whether a taxa or character is significantly more compositionally heterogeneous, improving usability.

The normalisation constants applied to form nRCFV address the key biases of the original RCFV—as the number of positions and taxa become larger, the contribution of the constant becomes smaller, thus resulting in a levelled curve that should resolve the core problem of RCFV—that it is less accurate and reliable at lower values of these factors than at higher values. Moreover, the plot of the RCFV values in relation to the number of taxa indicates that RCFV may be unreliable and inconsistent with fewer taxa, potentially both finding compositional heterogeneity where none exists and missing existing real heterogeneity as the final value is heavily influenced by taxon number, but not necessarily directionally.

### Why are RCFV and nRCFV not affected by missing data?

To observe the effect of missing data on RCFV, we took six real datasets with increasing amounts of missing data, and simulated 100 mimics of these datasets under two conditions, one retaining the proportion of missing data, and another filling in all gaps with characters. Notably, both RCFV and nRCFV values do slightly increase on average in the presence of missing data compared to no missing data. However, when amino acid frequencies were observed between the gapless and gapped simulations, an average frequency change of 0.0013 was observed, suggesting that, for example, the increase of 0.00009 in nRCFV is likely due to the real terms increase in compositional heterogeneity caused by “filling in” missing data in the simulated alignments. Comparing across different degrees of missing data, this difference between the datasets with and without missing data remains more or less the same and no correlation to the increasing level of missing data can be observed: Hence, missing data itself does not introduce a bias in the RCFV calculation, which needs to be accounted for.

The reason for this lack of bias is due to the original RCFV calculation, expressed in Eq. 1. By comparing mean frequency of a given taxon against the mean frequency across the entire dataset, RCFV indirectly accounts for gap characters by excluding them from the calculation. This allows the real compositional homogeneity to govern the calculation of the metric, rather than the distribution of missing data. Our results also show that the normalization model does not introduce an impact of missing data either. This is not surprising, as the normalization does not include any factors which would be directly influenced by the degree of missing data. Number of positions, taxa and character states are constant even if the amount of missing data changes within a dataset.

### Comparisons versus RCFV: a changing philosophy

This change within the calculation of nRCFV, however, removes an advantage of the non-normalized RCFV value: that the RCFV value for a group of taxa can be calculated from the sum of the tsRCFV of the taxa belonging to this group [23]. The same applies to groups of characters, which could be calculated by summing up the corresponding csRCFV values. For example, csRCFV values that are combinations of amino acids (e.g., hydrophobic amino acids) or nucleotides (e.g., AT), could be summed up over the corresponding amino acids (e.g., A, W, M, I, L, F and P) or nucleotides (e.g., A and T).

With the normalized values, this is no longer possible. For csRCFV, the normalised value can be calculated by summing the relative frequencies of the included states, calculating the RCFV of the sum, and then applying Eq. 6 to the csRCFV value for normalization. For groups of taxa no such solution is possible: to calculate the nRCFV for a group in this way, datasets should be reduced to the corresponding taxa and individually submitted to RCFV_Reader. As the new normalized value is independent of the number of taxa, the nRCFV for each of these sub-datasets can be directly compared to the nRCFV value of the entire dataset and to those of other monophyletic clades in the same manner.

### The effect of nRCFV on data selection and topology

Our analyses of empirical data show that the effect of the number of positions and taxa per gene has a strong impact on the selection of genes supposedly affected by compositional heterogeneity based on the RCFV value only. The percentage of shared genes is relatively low between RCFV and nRCFV with a maximum of 56% shared genes, but usually values < 40%. This suggests that, whilst RCFV could detect genes with similar levels of compositional heterogeneity to some degree, the other factors influencing the RCFV calculation also have an impact on data selection. The total alignment length of sextiles selected based on nRCFV values and lower compositional heterogeneity were substantially shorter (up to about 27.5%) than in the corresponding sextile based on RCFV values. This shows that shorter sequences were notably less favored by the RCFV metric, as our simulation analyses would suggest. On the other hand, the nRCFV values of the compiled datasets in Fig. 5 also showed that selection of genes based on RCFV did result in reduced compositional heterogeneity: albeit to a lesser extent than nRCFV. Hence, previous studies using RCFV remain valid to some degree.

However, it does appear that these slight differences in selection have a notable effect on topology. The RF distances from the complete tree were substantially higher in the selection of genes based on nRCFV than on RCFV, while it was the opposite with respect to degree of heterogeneity in the corresponding compiled datasets. This suggests that more compositionally heterogeneous genes are incorporated into the dataset in the lower sextiles using RCFV and accordingly the topology is more similar to the complete one exhibiting the maximal heterogeneity in the dataset. Hence, topologies based on nRCFV-selected genes seem to favour notably different optima within the likelihood landscape in contrast to those selected by RCFV, potentially suggesting that the real data is heavily affected by compositional heterogeneity. The RCFV and nRCFV selected sextiles do not converge on the same RF distance from the complete tree until they share 78.7% of their datasets—at 423 genes. Prior to this, they produce phylogenies from very different optima in the likelihood landscape. This does suggest a significant difference between the results of the two metrics.

### Alternative approaches to statistical measurement to deal with compositional heterogeneity

Besides the RCFV approach employing a statistical measurement, alternative methods have been suggested and used to deal with compositional heterogeneity. Like the RCFV approach, one group of tools allows exploration of compositional heterogeneity prior to any phylogenetic reconstruction. A variant of the χ^2^-test, which compares the homogeneity of the bases across sequences, is one such method (for review see [7]). It is implemented in programs such as PAUP, Tree-Puzzle and IQ-Tree [37, 39, 40]. While this test points out problematic sequences, comparison of heterogeneity across genes and partitions, and outside of the dataset being tested at the current time, is not straightforward.

Another way to address compositional heterogeneity is through a matched-pair test type metrics, currently encountered primarily in the form of the Maximum Symmetry test [8, 17, 19]. Maximum Symmetry Tests use the application of three matched-pairs tests of symmetry to assess both stationarity (i.e., compositional homogeneity) and homogeneity of substitution rates [8, 19]. This manages to evade the primary concern of matched-pair tests between all possible pairs in a dataset—a large amount of computational work that increases exponentially as the dataset expands. Instead, the Maximum Symmetry test compares only the two most divergent sequences to one another to assess whether they pass or fail the test. This method is implemented in IQtree [19, 37]. Due to the comparison of only the two most divergent sequences, it risks generating both false positives and negatives. One test might fail only due to the presence of a single outlying sequence in the entire partition. However, that single outlier might not in itself necessarily cause such a strong violation of the model assumptions that the phylogenetic reconstruction will be misled. Hence, a dataset is rejected as not being homogenous even though it is when taken as a whole. In addition, compositional heterogeneity is not necessarily linked to evolutionary rate [12, 14]. Hence, even though the pair of the two most divergent sequences do not show signs of compositional heterogeneity, it is not given that this is true for all of the sequences in these partitions. While the χ^2^-test does not allow comparison across genes, partitions and datasets, the matched-pair test is inefficient across large datasets and the Maximum Symmetry test has a high chance of produce false positives, nRCFV and ntsRCFV values allow for direct comparisons across different partitions, genes, datasets and taxa with low computational effort. On the other hand, other compositional heterogeneity measures provide direct significance test values as the results, whereas significance tests must be conducted for nRCFV and ntsRCFV values in addition and are not provided as standard.

A second group of methods applies sophisticated modelling approaches such as those employed by the CAT family of models [18, 41–43], or data recoding to reduce compositional heterogeneity originating from synonymous or quasi-synonymous substitutions [44, 45]. Although these site-specific models treat columns as statements of homogeneity, average frequency perspective of nRCFV or RCFV values can nonetheless be relevant due the application of Q matrices—and by extension equilibrium frequencies—to different categories, albeit to a much lesser degree than with other models. Furthermore, in these cases, nRCFV values can be used in combination with other tests to provide a double layer of protection. For example, the subsampling of large phylogenomic datasets to make them applicable for analyses using computationally intensive CAT models can be guided by nRCFV values. This has also the advantage that the selected genes or partitions will be more compositionally homogenous. In the case of recoding, ncsRCFV values can provide insights into individual character state heterogeneity to provide guidance for recoding strategies.

## Conclusions

RCFV is unique and highly useful in that it remains a highly convenient, low-cost method to detect compositional heterogeneity within a dataset, is one of the few methods of compositional heterogeneity detection that can be employed prior to phylogenetic analysis and allows assessment of genes, partitions and taxa with the same statistical measurement. As RCFV was itself derived from RCV and attempted to overcome some of its limitations through the addition of relative frequencies rather than occurrence counts of amino acids and nucleotides, so nRCFV represents the next evolution of this metric. This normalised relative frequency measure has statistical power across a wide variety of datasets both large and small, and in the future, with its current implementation in RCFV_Reader, is more convenient and computationally efficient than ever before, running on even large datasets in less than a minute on a local computer. In this manner, nRCFV can act as a useful screening method for these kind of phylogenetic artefacts for years to come.

## Data Availability

RCFV_Reader, which implements nRCFV, ncsRCFV and ntsRCFV value calculation is available at https://github.com/JFFleming/RCFV_Reader. Raw simulation data and real data is available at DataDryad at https://doi.org/10.5061/dryad.wpzgmsbpn.

## References

[1] Smith SD, Pennell MW, Dunn CW, Edwards SV (2020). Phylogenetics is the new genetics (for most of biodiversity). Trends Ecol Evol.

[2] Espinosa de los Monteros A (2020). Phylogenetics and systematics in a nutshell. Avian Malar Relat Parasites Trop Ecol Evol Syst.

[3] Sullivan J, Joyce P (2005). Model selection in phylogenetics. Annu Rev Ecol Evol Syst.

[4] Posada D, Buckley TR (2004). Model selection and model averaging in phylogenetics: advantages of Akaike information criterion and Bayesian approaches over likelihood ratio tests. Syst Biol.

[5] Whelan S, Goldman N (2001). A general empirical model of protein evolution derived from multiple protein families using a maximum-likelihood approach. Mol Biol Evol.

[6] Foster PG (2004). Modeling compositional heterogeneity. Syst Biol.

[7] Jermiin LS, Ho SY, Ababneh F, Robinson J, Larkum AW (2004). The biasing effect of compositional heterogeneity on phylogenetic estimates may be underestimated. Syst Biol.

[8] Jermiin LS, Jayaswal V, Ababneh FM, Robinson J (2017). Identifying optimal models of evolution. Bioinform Vol I Data Seq Anal Evol.

[9] Foster PG, Hickey DA (1999). Compositional bias may affect both DNA-based and protein-based phylogenetic reconstructions. J Mol Evol.

[10] Ho SY, Jermiin LS (2004). Tracing the decay of the historical signal in biological sequence data. Syst Biol.

[11] Nesnidal MP, Helmkampf M, Bruchhaus I, Hausdorf B (2010). Compositional heterogeneity and phylogenomic inference of metazoan relationships. Mol Biol Evol.

[12] Struck TH, Wey-Fabrizius AR, Golombek A, Hering L, Weigert A, Bleidorn C, Klebow S, Iakovenko N, Hausdorf B, Petersen M (2014). Platyzoan paraphyly based on phylogenomic data supports a noncoelomate ancestry of Spiralia. Mol Biol Evol.

[13] Pisani D, Pett W, Dohrmann M, Feuda R, Rota-Stabelli O, Philippe H, Lartillot N, Wörheide G (2015). Genomic data do not support comb jellies as the sister group to all other animals. Proc Natl Acad Sci.

[14] Kocot KM, Struck TH, Merkel J, Waits DS, Todt C, Brannock PM, Weese DA, Cannon JT, Moroz LL, Lieb B (2017). Phylogenomics of Lophotrochozoa with consideration of systematic error. Syst Biol.

[15] Martijn J, Vosseberg J, Guy L, Offre P, Ettema TJ (2018). Deep mitochondrial origin outside the sampled alphaproteobacteria. Nature.

[16] Fleming JF, Feuda R, Roberts NW, Pisani D (2020). A novel approach to investigate the effect of tree reconstruction artifacts in single-gene analysis clarifies opsin evolution in nonbilaterian metazoans. Genome Biol Evol.

[17] Ababneh F, Jermiin LS, Ma C, Robinson J (2006). Matched-pairs tests of homogeneity with applications to homologous nucleotide sequences. Bioinformatics.

[18] Lartillot N, Brinkmann H, Philippe H (2007). Suppression of long-branch attraction artefacts in the animal phylogeny using a site-heterogeneous model. BMC Evol Biol.

[19] Naser-Khdour S, Minh BQ, Zhang W, Stone EA, Lanfear R (2019). The prevalence and impact of model violations in phylogenetic analysis. Genome Biol Evol.

[20] Phillips MJ, Penny D (2003). The root of the mammalian tree inferred from whole mitochondrial genomes. Mol Phylogenet Evol.

[21] von Haeseler A, Janke A, Pääbo S (1993). Molecular phylogenetics. Verhandlungen der Deutschen Zoologischen Gesellschaft Proc German Zool Soc.

[22] Zhong M, Hansen B, Nesnidal M, Golombek A, Halanych KM, Struck TH (2011). Detecting the symplesiomorphy trap: a multigene phylogenetic analysis of terebelliform annelids. BMC Evol Biol.

[23] Kück P, Struck TH (2014). BaCoCa – a heuristic software tool for the parallel assessment of sequence biases in hundreds of gene and taxon partitions. Mol Phylogenet Evol.

[24] Whelan NV, Kocot KM, Moroz TP, Mukherjee K, Williams P, Paulay G, Moroz LL, Halanych KM (2017). Ctenophore relationships and their placement as the sister group to all other animals. Nat Ecol Evol.

[25] Vasilikopoulos A, Misof B, Meusemann K, Lieberz D, Flouri T, Beutel RG, Niehuis O, Wappler T, Rust J, Peters RS (2020). An integrative phylogenomic approach to elucidate the evolutionary history and divergence times of Neuropterida (Insecta: Holometabola). BMC Evol Biol.

[26] Laumer CE, Gruber-Vodicka H, Hadfield MG, Pearse VB, Riesgo A, Marioni JC, Giribet G (2018). Support for a clade of Placozoa and Cnidaria in genes with minimal compositional bias. Elife.

[27] Wang Y, Zhang R, Ma Y, Li J, Fan F, Liu X, Yang D (2021). Low-coverage whole genomes reveal the higher phylogeny of green lacewings. Insects.

[28] Cerca J, Rivera-Colón AG, Ferreira MS, Ravinet M, Nowak MD, Catchen JM, Struck TH (2021). Incomplete lineage sorting and ancient admixture, and speciation without morphological change in ghost-worm cryptic species. PeerJ.

[29] Li J, Lemer S, Kirkendale L, Bieler R, Cavanaugh C, Giribet G (2020). Shedding light: a phylotranscriptomic perspective illuminates the origin of photosymbiosis in marine bivalves. BMC Evol Biol.

[30] Lemmon EM, Lemmon AR (2013). High-throughput genomic data in systematics and phylogenetics. Annu Rev Ecol Evol Syst.

[31] Lozano-Fernandez J (2022). A practical guide to design and assess a phylogenomic study. Genome Biol Evol.

[32] Lartillot N, Philippe H (2004). A Bayesian mixture model for across-site heterogeneities in the amino-acid replacement process. Mol Biol Evol.

[33] Tavaré S (1986). Some probabilistic and statistical problems in the analysis of DNA sequences. Lect Math Life Sci (Am Math Soc).

[34] Rambaut A, Grass NC (1997). Seq-Gen: an application for the Monte Carlo simulation of DNA sequence evolution along phylogenetic trees. Bioinformatics.

[35] Schliep KP (2011). phangorn: phylogenetic analysis in R. Bioinformatics.

[36] Paradis E (2012). Analysis of phylogenetics and evolution with R.

[37] Minh BQ, Schmidt HA, Chernomor O, Schrempf D, Woodhams MD, Von Haeseler A, Lanfear R (2020). IQ-TREE 2: new models and efficient methods for phylogenetic inference in the genomic era. Mol Biol Evol.

[38] Ly-Trong N, Naser-Khdour S, Lanfear R, Minh BQ (2022). Alisim: a fast and versatile phylogenetic sequence simulator for the genomic era. Mol Biol Evol.

[39] Wilgenbusch JC, Swofford D (2003). Inferring evolutionary trees with PAUP. Curr Protoc Bioinform.

[40] Schmidt HA, von Haeseler A (2007). Maximum-likelihood analysis using TREE-PUZZLE. Curr Protoc Bioinform.

[41] Si Quang L, Gascuel O, Lartillot N (2008). Empirical profile mixture models for phylogenetic reconstruction. Bioinformatics.

[42] Höhna S, Landis MJ, Heath TA (2017). Phylogenetic inference using RevBayes. Curr Protoc Bioinform.

[43] Schrempf D, Lartillot N, Szöllősi G (2020). Scalable empirical mixture models that account for across-site compositional heterogeneity. Mol Biol Evol.

[44] Phillips MJ, Delsuc F, Penny D (2004). Genome-scale phylogeny and the detection of systematic biases. Mol Biol Evol.

[45] Ishikawa SA, Inagaki Y, Hashimoto T (2012). RY-coding and non-homogeneous models can ameliorate the maximum-likelihood inferences from nucleotide sequence data with parallel compositional heterogeneity. Evolut Bioinform.

